# Bonding strength performance of bamboo-based composite materials: An in-depth insight for sustainable construction applications

**DOI:** 10.1016/j.heliyon.2024.e32155

**Published:** 2024-06-03

**Authors:** Yousef Sewar, Mugahed Amran, Siva Avudaiappan, Yaser Gamil, Raizal S.M. Rashid

**Affiliations:** a Civil Engineering Department, Faculty of Engineering, Thamar University, 9676, Thamar, Yemen; b Department of Civil Engineering, College of Engineering, Prince Sattam Bin Abdulaziz University, 11942, Alkharj, Saudi Arabia; c Department of Civil Engineering, Faculty of Engineering and IT, Amran University, 9677, Amran, Yemen; d Departamento de Ciencias de la Construcción, Facultad de Ciencias de la Construcción y Ordenamiento Territorial Universidad Tecnológica Metropolitana, Santiago, Chile; e Department of Civil, Environmental and Natural Resources Engineering, Luleå University of Technology, Sweden; f Department of Civil Eng., School of Eng., Monash University Malaysia, Jalan Lagoon Selatan, 47500, Sunway, Selangor, Malaysia; g Department of Civil Engineering, Universiti Putra Malaysia, 43400, Serdang, Selangor, Malaysia

**Keywords:** Bonding strength, Performance, Biomaterial, Bamboo-based composite materials

## Abstract

This review systematically examines the multitude of factors influencing bonding strength in bamboo-based composite materials, given the rising prominence of bamboo as a green building material. With bamboo's inherent variability in mechanical properties and structure, engineered bamboo products have emerged to address challenges related to connections and joints. Such advancements have necessitated a detailed exploration of adhesive systems, a significant cost determinant in bamboo production. The adhesive bonding mechanism in bamboo, akin to wood, involves intricate processes including adhesive spreading, penetration, and solidification, influenced by the unique chemical composition of bamboo. The interfacial bond quality plays a pivotal role in determining the durability and performance of the final products, with numerous factors such as bamboo species, layered structure, adhesive type, and treatment types impacting the mechanical properties. Particular attention is given to the disparities in physical and mechanical properties between the bamboo culm's core and shell layers, attributing complexities to the gluing process. Examining shear failure strength reveals its criticality in mechanical investigations, with variations in bonding strength affecting the outcome. The review underscores the need for consistent quality control and adept manipulation of these influential factors for the successful manufacture of bamboo-based products. A comprehensive discussion ensues on the variables controlling the bonding properties of the developed bamboo products, aiming to evaluate and highlight the optimal parameters and procedures essential for enhancing the quality and reliability of bamboo-based composite materials for sustainable construction applications.

**Table:** Abbreviation

B-bambos	Bambusa Bambos.	LMWPF	Low Molecular Weight Phenol Formaldehyde.
BFRC	Bamboo-based Fiber Reinforced Composite.	NafKf	Neosinocalamas affinins (Rendle) keng f.
BL	Bamboo Laminate.	Phe	Phyllostachs heterocycle.
BLS	Bambusa Laminates Strips.	UPF	Urea Phenol Formaldehyde.
BPLS	Bmaboo Parallel Strand Lumber.	Dfa	Dendrocalamus farinosus.
CCLT	Composite Cross-Laminated Timber.	BLVL	Laminated Bamboo-bundle Veneer Lumber.
DA	Dendrocalomus Asper.	OBFMs	Oriented Bamboo Fiber Mats.
DSer	Dendrocalamus Sericeus.	CLB	Cross Laminated Bamboo.
E	Parallel to Grain.	P.u K	Poplar (Populus ussuriensis Kom) wood.
E.S	Erythrophleum Suaveolens.	BLT	Bleaching Treatment.
GanB	Guadua angustifolia Bamboo.	I	Internode
GLevis	Gigantochloa Levis.	TFR	Tannim resorcinol Formaldehyde.
GSr	Gigantochloa Scortechinii.	EPI	Polymer Isocyanate.
GLGB	Glued Laminated Guadua Bamboo.	OLBL	Overlaid Laminated Bamboo Lumber.
H	Cross-Laminated Horizontally.	BWHC	Reclaimed Bamboo Chopsticks-wood veneers Hybrid Laminated Composite.
HP	Hot Press.	PVA	Polyvinyl Acitate.
I–I	Inner-Inner.	RPF	Resorcinol Phenol Formaldehyde.
LBB	Loose Bamboo Bundle.	EMDI	Emulsion Methyldiisocyanate.
LBL	Laminated Bamboo Lumber	BZB	Bamboo Zephyr Boards.
MF	Melamine Formaldehyde.	WV	Wood Veneer.
MUF	Melamine Urea Formaldehyde.	BC	Bamboo Chopstick
OI	Outer-Inner	Soy	Soy-Flour-Based Adhesive.
OO	Outer-Outer.	HPA	Hypird Polymer Adhesive.
P	Perpendicular to Grain.	LU	lay-up of BWHC as in figure (.).
PBfY	Phyllostachys bambusoides f.shouzhu Yi.	PPM	Phyllostachys Pubescens Mazel.
PF	Phenol Formaldehyde.	PP	Phyllostachys Pubescens.
PlyB	Plybamboo.	BS	Bamboo Scrimber.
PUR	Polyurethane.	Phe	Phyllostachys heterocycle.
UF	Urea Formaldehyde.	OBFRC	Outdoor Bamboo Fiber Reinforced Composite.
UN	Untreated.	CAT	Caramelization Treatment.
V	Cross-Laminated Vertically.	LT	Laboratory Temperature.
VPD	Vacuum Pressure Soak.	PPM	Phyllostachys Pubescens Mazel.
CLBT	Cross-Laminated Flattened Bamboo and Timber.	CLB	Cross Laminated Bamboo.
WFP	Wood Failure Percentage		

## 1 Introduction

Historically, wood and bamboo have been essential materials in global construction, due to continuous advancements in structural technology [[1], [2], [3], [4], [5], [6], [7]]. Bamboo, however, poses challenges due to its inconsistent mechanical qualities and variable geometry and structure. Its irregular tube shape and diameter complicate joint and connection formation. As a result, the industry has pivoted towards creating sturdier, engineered bamboo products like bamboo lumber, laminated bamboo [[8], [9], [10], [11], [12]], and bamboo scrimber [[13], [14], [15], [16], [17]], bamboo-timber composite [18,19]. This is in addition to developments in particleboard [20,21], medium-density fiberboard [2], and oriented strand board [22]. It's worth noting that alterations in raw material can impact the final product's characteristics, necessitating adjustments in treatment processes, such as the adhesive system.

Considering the substantial cost that adhesives contribute to bamboo product manufacturing, a thorough examination of the correlation between bamboo's bonding strength and its compatibility with composites is crucial for the material's future in the industry [23]. Bamboo can also be integrated with other prevalent structural materials like reinforced concrete and timber. A considerable amount of research has been conducted by both academics and industry professionals to unravel the intricacies of wood bonding mechanisms and theories [[24], [25], [26], [27], [28]]. Marra et al. [28] identified various interconnected factors involved in wood bonding, including surface wetting with adhesive, adhesive spreading, penetration, cellular alteration, and solidification. Given that bamboo is a lignocellulosic material with a chemical makeup similar to wood, the principles governing wood bonding are likely applicable to bamboo, albeit with certain fundamental distinctions that could affect the bonding process in bamboo [[29], [30], [31], [32], [33]].

The durability and quality of products are significantly influenced by the interfacial bond in both bamboo [29,34] and bamboo composites [35], playing a critical role in the overall performance of the final product [[36], [37], [38]]. Factors such as adhesive penetration, curing rate, degree of adhesion, clamping pressure, and clamping time all contribute to the strength of the interfacial bond [18,39,40]. Proper bonding ensures efficient stress distribution across fiber bundles when the product is subjected to loading, thus enhancing the engineering properties of the composite [41]. Several variables, including soil and site conditions, bamboo species, age of the culm, and element size, can influence the engineering properties, as well as the bonding strength [35,[42], [43], [44], [45], [46], [47], [48], [49], [50]]. Furthermore, the bamboo species, layered structure, glue spread rate, adhesive type, resin content, strip arrangement, density, and treatment types all function a vital character in determining the mechanical properties of bamboo composite [[51], [52], [53], [54], [55], [56]]. It's worth noting that weak interfacial connection between the shell layer and the core can significantly undermine the mechanical properties of core-shell structured BPC [57].

**Fig. 1:**
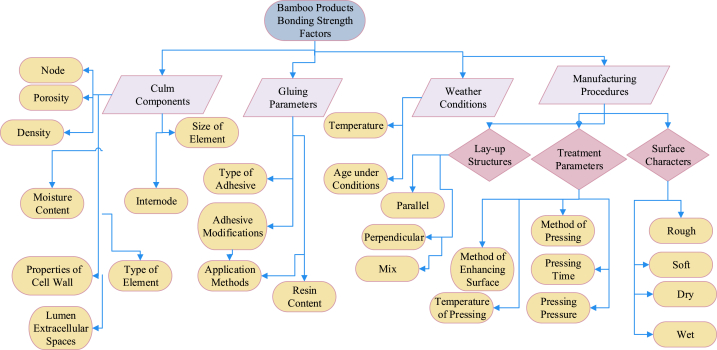
Factors affecting on bonding strength of bamboo products [34,54,[61], [62], [63], [64], [65], [66], [67], [68]].

The engineering properties of a bamboo culm's main body differ significantly from its inner and outer layers, which contain wax and silicium compounds, complicating the gluing process [58]. Tsujino et al. [59] identified shear failure strength as a vital factor in their mechanical model, with varying limiting values based on bonding strength. A decrease in effective pressure within gap regions resulted in reduced bonding strength [60]. Ensuring consistent quality control in lumber production and careful management of these critical factors is integral for the successful manufacture of bamboo-based products. This comprehensive review focuses on discussing the variables that impact the bonding properties of newly developed bamboo products, as identified through extensive literature review. The aim of this review is to evaluate bonding performance, highlight influential factors affecting on the bonding quality, and identify the optimal resin percentages, types of adhesive, bamboo species, and pressing, methods of treatment, and laying up, and procedures of manufacturing. These findings will contribute to high-quality, reliable bamboo-based construction applications. Table 1 summarizes the impact of various factors on the engineering properties of bamboo products, particularly bonding, as reported by researchers globally. The factors affecting bonding strength in bamboo products can be broadly categorized into four groups: (i) culm components, (ii) gluing parameters, (iii) weather conditions, and (iv) manufacturing procedures. Fig. 1 provides a detailed summary of specific factors under these four categories.

**Table 1:**
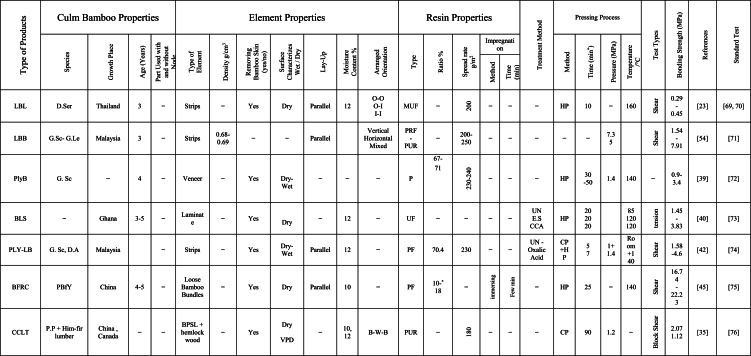

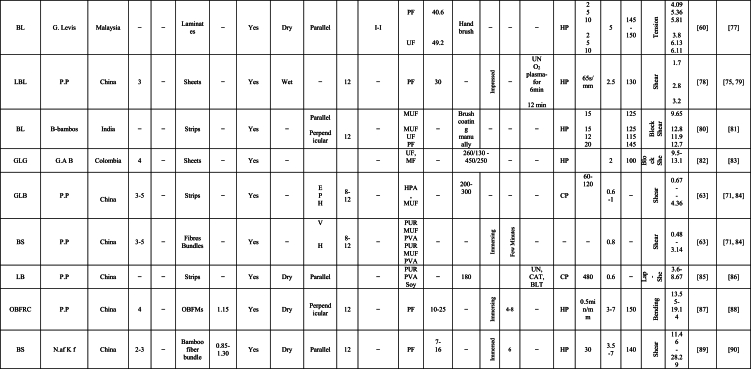

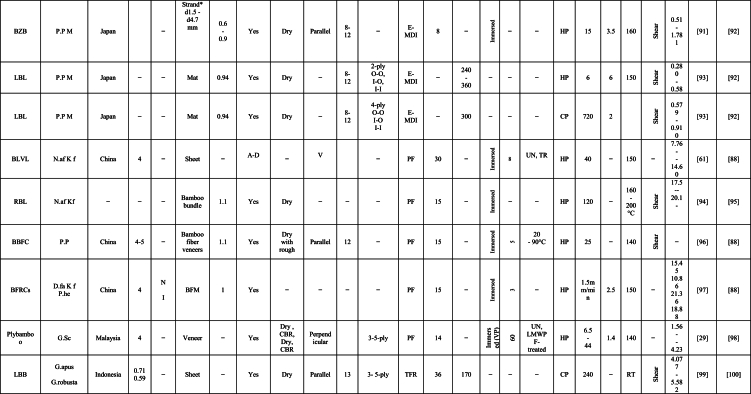

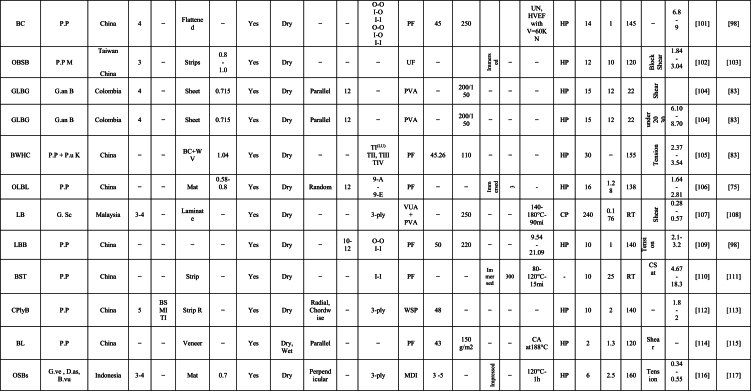
Summary the influence of several factors on bonding property of bamboo-based construction applications as reported by many researchers worldwide [23,29,35,39,40,42,45,54,60,61,63,69,[70], [71], [72], [73], [74], [75], [76], [77], [78], [79], [80], [81], [82], [83], [84], [85], [86], [87], [88], [89], [90], [91], [92], [93], [94], [95], [96], [97], [98], [99], [100], [101], [102], [103], [104], [105], [106], [107], [108], [109], [110], [111], [112], [113], [114], [115], [116], [117]].

## 2 Effect of gluing factors on bonding strength

The bonding performance of bamboo-based construction applications can be significantly influenced by the application of gluing factors. Scientifically, the intrinsic properties of bamboo, such as its high silica content, variable moisture content, and anisotropic nature, pose challenges for achieving consistent and durable adhesive bonds. Gluing factors, including the type of adhesive used, the preparation of bamboo surface, and the curing conditions, play critical roles in determining the bond quality. Specifically, selecting an adhesive that is compatible with bamboo's chemistry, ensuring a clean and well-prepared surface free from contaminants, and optimizing curing conditions (temperature, pressure, and time) can enhance the penetration of adhesive, improve wetting, and promote better mechanical interlocking. Thus, careful optimization of these gluing factors is imperative to maximize the structural integrity and longevity of bamboo using in construction applications. In brief, the complexity of bamboo's structure and properties demands a holistic approach to bonding. A symbiotic relationship between the bamboo's characteristics, the adhesive's properties, and the bonding process is necessary to achieve the best results in bamboo-based construction applications.

Furthermore, the bonding strength and quality of bamboo-based composite is affected by several parameters. One of this parameters is the gluing factors such as type of adhesive, solid content of adhesive, spread rate of adhesion, application methods of resin, and adhesive modifications. In this section, the gluing parameters are discussed according to literature review results and data. Table 1. Summary of many papers that explain the factors on bonding strength of bamboo products.

### 2.1 Adhesive type, modifications and resin content on bonding strength

The mechanical integrity of bamboo-based products is significantly influenced through the choice of adhesive utilized. To ascertain the most efficacious adhesive variant and its optimal resin content for bamboo applications, an analysis of existing research is imperative. Distinct disparities in bonding strength are predominantly ascribed to the type of adhesive employed. In an experiment conducted on glued laminated Guadua bamboo (GLG), four adhesive types were tested for bond shear strength, with the application rates adhering to manufacturer guidelines and recommendations [118]. Of the adhesives assessed, the melamine-urea-formaldehyde (MUF) bond shear strength emerged as superior, though the differences among the four adhesives were marginal. Moreover, Alipon et al. [62] explored the bond shear strength of manufactured bamboo boards (BB) employing six different adhesives. PVAc adhesive was utilized to examine the interface of bamboo boards in both indoor and outdoor construction settings. In contrast, the remaining five adhesives were evaluated solely for indoor applications. Findings revealed that PVAc exhibited the highest bonding strength in both external (5.68 MPa) and internal (5.15 MPa) environments. However, these values were modest compared to the Polyvinyl Alcohol (PVA) bonding strength recorded for laminated bamboo as documented in study [85].

Bansal et al. [80] conducted an investigation into the bonding strength of laminated bamboo derived from Bambusa bamboos, utilizing three distinct adhesive types. The findings from this study revealed that laminates bonded with Phenol-Formaldehyde (PF) are appropriate for external applications, while those bonded with (UF) and (MUF) are more suited for internal use. Correal et al. [82] echoed these results in their research.In a separate study, it is also scrutinized the bonding strength and durability of glued laminated Guadua bamboo, using four different adhesive types, all applied at rates recommended by their respective manufacturers. They discovered that the differences in bonding strengths among the various adhesive types were negligible. The researchers subsequently concluded that a mixture of 50 % melamine-formaldehyde and 50 % urea-formaldehyde is the optimal adhesive combination for construction applications of GLGB. The average bonding strength recorded for all four adhesives was 12.8 MPa, a value closely aligned with the bonding strength of Phyllostachys pubescens, which stands at 13.3 MPa [119]. Moreover, Xing et al. [63] utilized five different adhesives to evaluate the bonding shear strength of cross-laminated bamboo produced from bamboo scrimber and glued laminated bamboo. Their findings highlighted significant disparities in bonding shear strength, attributable to the type of adhesive utilized.

MUF adhesive demonstrated superior shear resistance, with values ranging from 2.53 to 5.36 MPa, when used for glued laminated bamboo specimens under various loading configurations [120,121]. In contrast, PVA exhibited remarkable consistency and is thus highly recommended for bonding bamboo scrimber in cross-laminated applications. Phenol formaldehyde (PF) is prominently utilized in the bamboo and wood processing industries for both interior and exterior applications, specifically for bamboo scrimber [120,121]. This preference is due to its advantageous properties, including robust dry bonding strength, affordability, and prevalence, constituting approximately 90 % of all wood adhesive applications [122]. As a thermosetting adhesive, PF is capable of withstanding curing temperatures exceeding 100 °C and offers exceptional bonding strength. In addition, global formaldehyde production is projected to increase by more than 2 % annually [123]. Wang et al. [124] assessed the bonding quality and durability of cross-laminated timber hemlock using (EPI) and (PUR) adhesives. The study found that PUR adhesive resulted in higher delamination rates during hem-fir CLT manufacturing, was more susceptible to pressure, and produced lower wood WFP compared to EPI adhesive. Furthermore, Shah et al. [85] conducted lap-joint shear tests on five commercially available adhesives: PAV, PU, RPF, Soy, and UPF. These adhesives were applied to various bamboo surface treatments. The findings indicated that resorcinol phenol formaldehyde (RPF) exhibited the highest bonding strength, reaching 8.5 MPa with bleached treatment, while polyurethane (PU) displayed a bonding strength of 8 MPa on untreated surfaces.

**Fig. 2:**
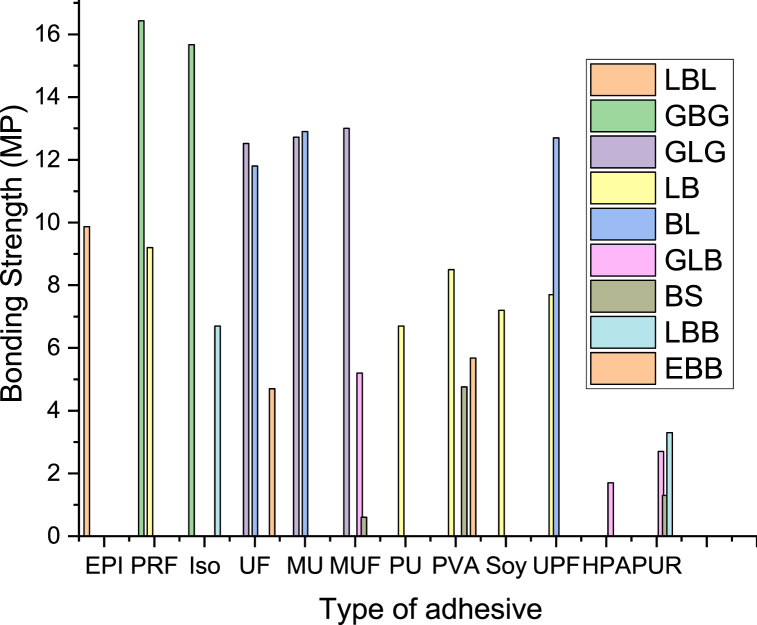
Influence of glue type on bonding strength of bamboo products: LBL and GBG [126]; GLG [118]; LB [85]; BL [80]; GLB and BS [63]; LBB [54]; EBB [62].

For instance, Zhang et al. [45] explored the bonding characteristics inherent in bamboo fiber-reinforced composites (BFRC). In their experiment, bundles of bamboo fibers were adhered together utilizing varying levels of resin content, and subsequently arranged in distinct combination patterns. The findings revealed that an augmentation in resin content directly correlated with an enhancement in bond quality for bamboo boards, as depicted in Fig. 2. Moreover, the bolstering of mechanical properties in bamboo boards, including elasticity modulus (MOE) and rupture modulus (MOR), can be ascribed to the heightened resin content situated along the edge of the bonding region. This increased resin presence is pivotal in bolstering the board's capacity to withstand external pressure, as corroborated by Refs. [39,44,45,125].

**Fig. 3:**
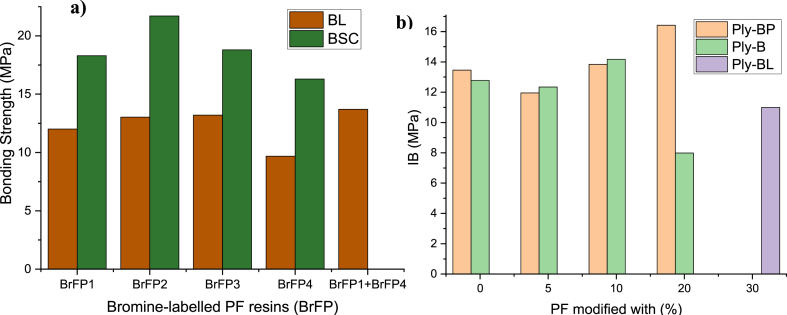
Bonding strength of bamboo products with diverse MWs of resin and twice-adhesive dispensing, BL [32]; BSC [129]. Notices: four differs molecular weights in bromine-labelled PF resin used as: BrPF1 = 542; BrPF2 = 945; BrPF3 = 1504; and BrPF4 = 1964 to 2001; at Ply-BP PF modified by PVA; at Ply-B PF adapted by (LMW)PF; at Ply-BL PF adapted by PTUF.

**Fig. 4:**
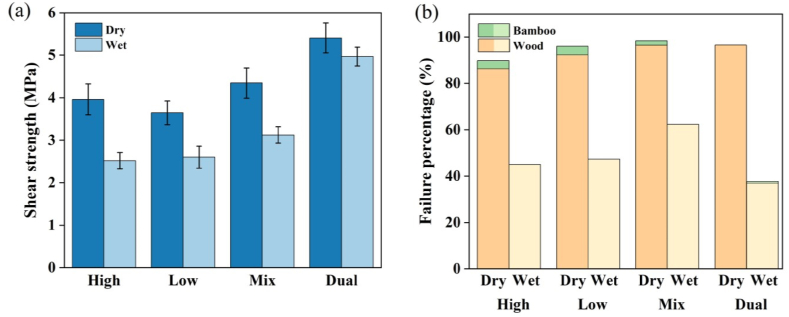
Bonding performance of solid bamboo-wood laminates using MWFP resin application methods and levels: (a) solid bamboo-wood bondline shear strength and (b) fiber failure percentage in dray and wet conditions. N = 30 for each column [114]. High MWFP, Low MWFP, Mix: mixture of High MWFP and Low MWFP, Dual MWFP: Low MWFP on bamboo surface and High MWFP on wood surface.

The molecular size of adhesive mechanisms plays a significant role in penetrating bamboo cells to form nano-interpenetrating polymer networks in wood products. Different studies have shown that low molecular PF resin is more suitable for forming mechanical interlocks. In contrast, high molecular PF resin is better for forming bond lines and their thickness and strength [32,34,89,127,128]. Zhang [32] applied different MWs of resin and twice adhesive dispensing on a bamboo interface to evaluate bonding shear strength (Fig. 3a). The lower bonding strength of BrPF4 was attributed to the starved bond line. The failure of the resin to penetrate BrFP4 to make a firm bond is due to most of the adhesive staying in the bond line, similar to results found by Ref. [129] (Fig. 3b). The bonding interlines recorded the peak bonding strength after twice adhesive dispensing, at 13.7 MPa, 42.7 %, and 13.2 % higher than BrFP4 and BrFP1, respectively. The study concluded that an acceptable bonding interface required high-MW resins to stay in the adhesive coating and lower penetration of MW resin into the bamboo middle to create an interpenetrating grid of polymer. Zhang [114] examined the effectiveness of resin application methods and levels on the bonding performance of structural bamboo-wood laminates. The outcomes demonstrated that the bonding shear strength achieved the highest in the Dual group, but the fiber failure percentage was lower than in the Mix, as shown in Fig. 4. In addition, some researchers investigated the effect of PF resin modified by different resin content levels. Guan [34] examined the influence of PF resin modified with varied levels of (LMW) PF on the bonding performance of two-ply bamboo. The results showed that the highest IB was achieved at 10 % LMW PF.

**Fig. 5:**
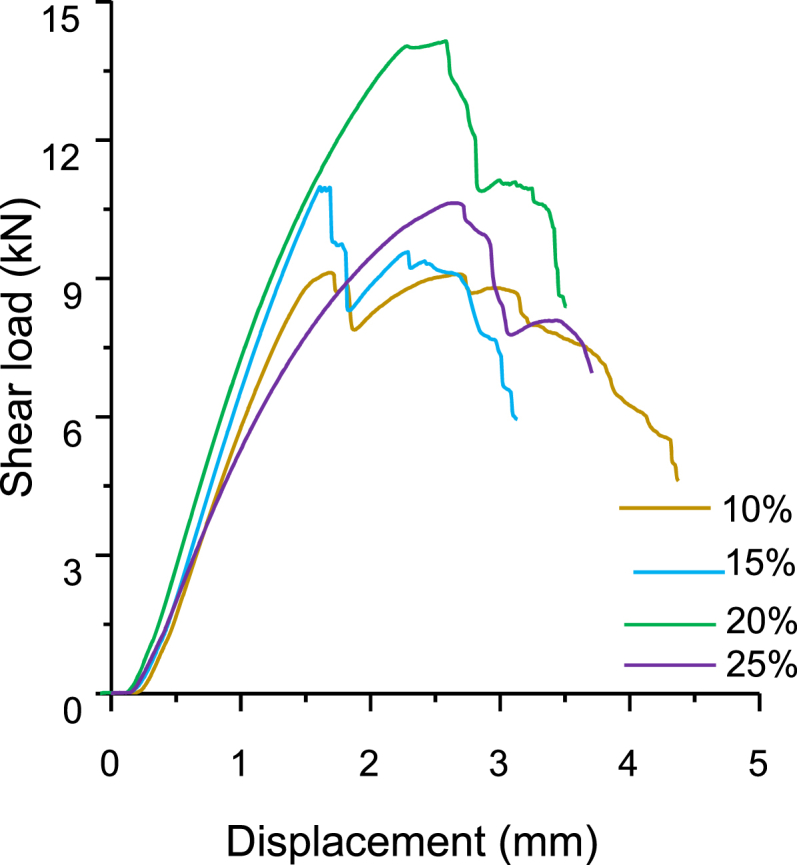
OBFRC shear force-displacement behaviour with different resin content [87].

**Fig. 6:**
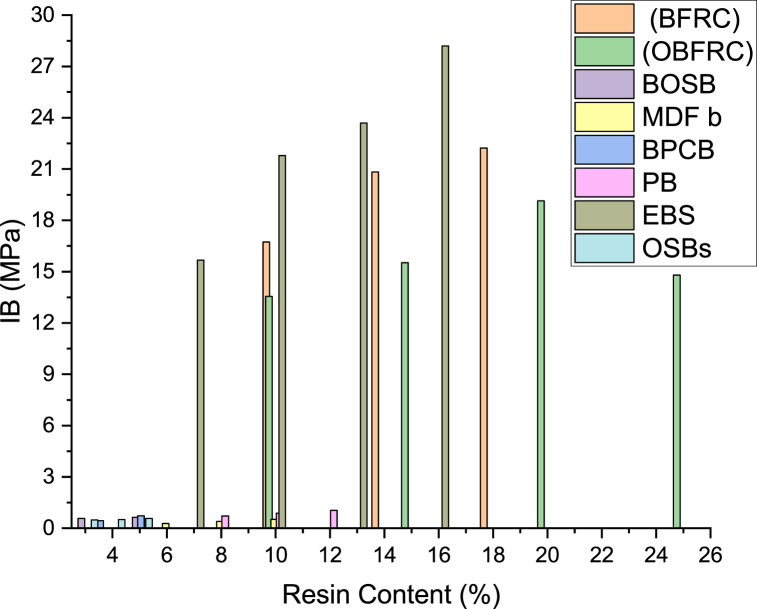
Shear strength of bamboo products, BFRC [45]; OBFRC [87]; BOSB [65]; MDF b [136]; BPCB [137]; PB [21]; EBS [89]; OSBs [116].

The increase in resin contented from 10 to 20 % gradually increased the bonding shear strength of OBFRC to 19.21 MPa [87]. However, increasing the resin content to 25 % resulted to a lessening in OBFRC shear strength to the levels of OBFRC 15 % and FRC-10 %. Therefore, effective mechanical bonding of OBFRC is negatively affected if resin content surpasses 20 %. OBFRC bonding interface failure under shear load is attributed to a decrease in OBFRC load in a sustained manner when resin content reaches a critical load (Fig. 5). The appearance of fewer and smaller cracks and the greater displacement of OBFRC samples before failure, is owing to the higher resin contented (20 and 25 %). The bonding strength of bamboo scrimber quality improved with the increase in PF resin loading [130,131]. Fauzi et al. [116] investigated the impact of resin content on the internal bonding strength of oriented strand boards (OSBs) made from different bamboo species using resin contents of 3, 4, and 5 %. The results demonstrated that the IB increases with the increase in resin content across all bamboo species. Similar results were noticed in OSBs manufactured from wood [[132], [133], [134], [135]]. Resin content significantly affects the gluability and strength of bamboo products (Fig. 6), where the bonding strength of bamboo products increased in the range between 10 and 20 % resin content. Based on the studies reviewed, adhesive type, quality, and quantity play a vital role in the quality of building bamboo composites, as they determine the interface and proper penetration between the lamina and the fiber. It can be concluded that PVAc and PRF have good bonding strength for exterior and interior applications, with the optimum resin content ranging from 10 to 18 % to achieve the best bonding strength.

### 2.2 Effect of glue spread rate on bonding strength

**Fig. 7:**
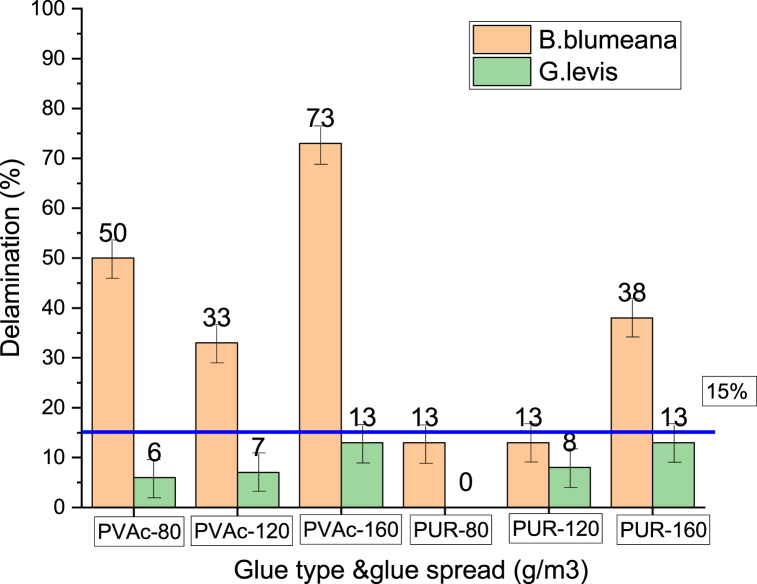
ALBL delamination percentage [8].

The spread rate of glue has a momentous influence on the mechanical behavior of bamboo and wood products. Most investigations on bamboo and its productions adhere to the spread rates previously determined by local adhesive manufacturers [39,99,104,119,[138], [139], [140]]. Juanito et al. [8] used ALBL produced from two bamboo species to investigate bond delamination, utilizing PVAc and PUR as adhesives. The resin spread rate was in accordance to the delamination test PNS [141], with different applied spread range of 80, 120, and 160 g/m2. The G. levis bamboo species did not exceed the 15 % blue horizontal line of the delaminated specimen, in any case of the adhesive kind and spread rate combination (Fig. 7). However, the blumeana bamboo species with PVAc did not pass the test regardless of the used spread rate. Two PUR adhesive spread rates, 80 and 120 g/cm2, passed the delamination test. The B. blumeana cutin layer, which passively impacted the quality of the surface preparation of the laminates, performed poorly in bonding. Compared to G. levis, the B. blumeana waxy cutin layer still looked smooth after sanding, which enhanced internal bonding. Based on the delamination tests and mechanical properties, the recommended spread rate for both bamboo species is 80 g/m^2^. This spread rate can be applied to ALBL because its mechanical and physical properties conform to the E. bamboo minimum strength requirement for general purposes, as per (DTI, BPS)-2015 [142].

**Fig. 8:**
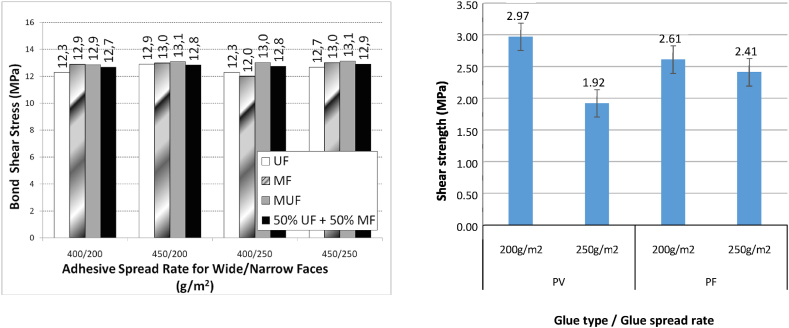
Influence of spread range on bonding shear strength of bamboo products: (a) Shear strength of GLGB [118]; (b) Shear strength of laminated bamboo timber [144].

It is used six spread rates of adhesive to carry out glue line tests in glued laminated Guadua bamboo using a 50 % melamine formaldehyde (MF) with urea-formaldehyde (UF) adhesive by Ref. [82]. Based on the bamboo failure percentage and bond shear strength, the best recommended spread rates of adhesive are 150 and 300 g/m^2^ on the narrow and wide faces, respectively. Nugroho and Ando [93] used three glue spread rates (240, 300, 360 g/m^2^) on three combinations of bamboo zephyr layered structural made of Mature Moso Bamboo. The best recommended spread rate of adhesive is 300 g/m2 for all structural combinations. The high internal bonding strength is attributed to the resin spread rate, but in two combinations, the variations between the glue spread rates of 300 and 360 g/m3 were not significant. Despite its less significant adhesive spread rate, the inner-inner arrangement tends to decrease after reaching 300 g/m^2^. Adewunmi et al. [143] used three different glue spread rates to determine the best quantity of glue for bamboo lamination. The spread rates were 150, 200, and 250 g/m^2^. To achieve better mechanical properties, they recommended an increased amount of glue to increase bonding strength. It can be safely consummated that the optimal spread rate is 200 g/m^2^ because, at this rate, all produced bamboo conforms to all technical specifications, as shown in Fig. 8a and b. All results demonstrated variation in bonding strength with the variation of spread rate that nearly depends on the type of bamboo species.

## 3 Effect of bamboo component on bonding strength

**Fig. 9:**
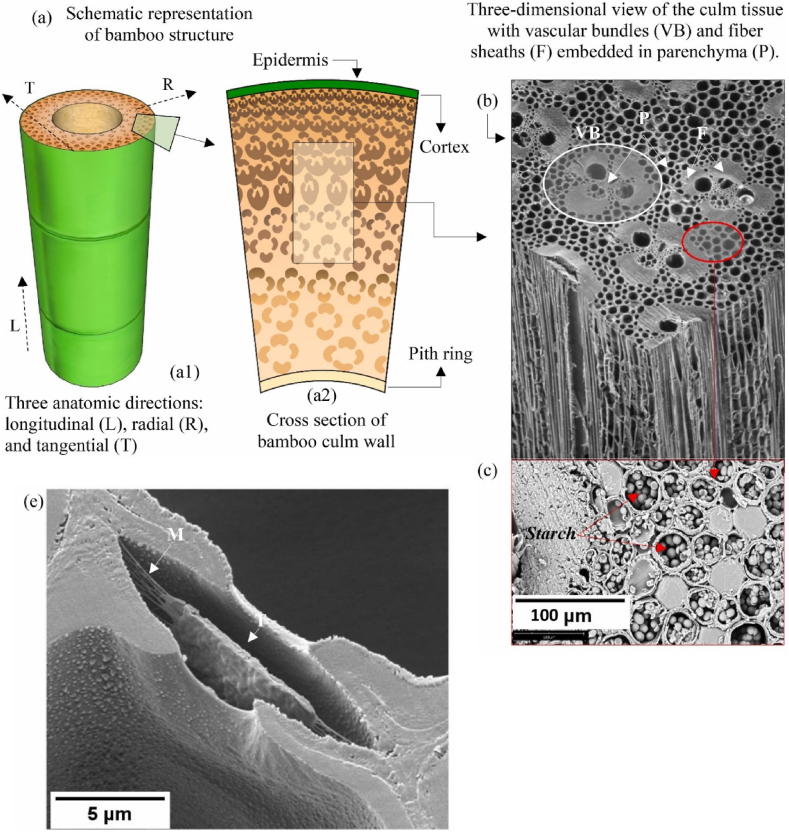
Microstructure of bamboo: (a) Schematic and (b) Moso bambooS′ SEM micrograph, (c) starch grain occlusions in Moso, (d) SEM of parenchyma cell walls of Moso present small pit member (PM) and polylamellate (a) [33] cell wall structure, (b) [157], (c) [33,114], (e) [158].

The bonding quality of bamboo products is also affected by the structural properties and basic physical of adherent bamboo, such as porosity, density, properties of cell wall, moisture content, lumen, extracellular spaces, internode, node, type of element, size of element. The parenchyma cells and bamboo fibers cells are the main compositions of bamboo culm [[146], [147], [148], [149], [145]]. Bamboo is uneven on microscopic structure and also is biomass material. The microstructure of bamboo is explained by different researchers as in Fig. 9ae.

### 3.1 Culms’ contents

The chemical content of bamboo culm, such as waxy and SiO2, can negatively affect the gluability of bamboo products [150]. The culm contains a siliceous at inner and siliceous and waxy at outer layers [151]. The weak bonding of adhesive and bamboo without surface treatment represents a challenging task in the construction of bamboo products [61,91,93,[152], [153], [154], [155], [156]]. This problem can be addressed by removing the inner and outer chemical components in layers during the manufacturing process of bamboo products. Failure to address this issue adequately will result in products that disappoint consumers and the industry due to the very weak surface appearance and interface bonding [91,93]. Furthermore, it requires a higher quantity of a suitable adhesive and sufficient pressing duration, consequently increasing production costs [23].

However, the drawbacks mentioned above do not mean a lack of studies using a whole of bamboo without removing the undesired components in the inner and outer culms. Pannipa et al. [23] studied the surface characteristics of LBL produced from D. sericeus with different layer structures. The outer-outer layer structure yielded the lowest bonding strength at 0.29 MPa. This is attributed to the chemical components, such as wax and silica, of the bamboo culm, which passively influenced the glue line bonding strength. The same findings were confirmed by Refs. [39,93].

To improve the utilization of whole bamboo resources, a new technology has emerged that involves roll-pressing bamboo at high temperatures to manufacture bamboo fiber-reinforced composites (BFRC). This technology utilizes the full bamboo culm as a manufacturing item, eliminating bonding drawbacks for both face of culm. As a result, the bamboo utilization ratio has increased to 90% [45,94,96,[159], [160], [161]]. Zhang and Yu [45] investigated the bonding strength of BFRC using this technique with different resin content levels. The results showed that an upsurge in resin contented to 18% resulted in a bonding strength of approximately 22.5 MPa, compared to about 16.5 MPa at 10% resin content. Glue ingesting due to the coarse surfaces of laminated bamboo bundle (LBB) is a significant problem in the manufacturing process of BFRC [29,162]. However, this technique has demonstrated acceptable bonding strength for applying the products in both exterior and interior structural applications.

To overcome bond issues, scraping or removal of undesirable siliceous and wax layers is preferable [163]. The only disadvantage of this method is its cost [93,163]. To mitigate the long-ranging effects of the chemical component in culms, different approaches can be adopted to improve the bonding strength of bamboo products. The traditional mechanical separation process is used to produce bamboo sliver, bamboo bundle, and bamboo strips [164,165]. To accomplish the requirements for the mechanical properties and bonding of bamboo products, the chemical component in an outside and inside layers of the culm are removed. However, the necessary process of removing unwanted substances has its disadvantages. It passively impacts the yield of bamboo-produced units and hinders the effective exploitation of bamboo [45,164]. To avoid the complications mentioned above, a crushing machine equipped with two different gears and several pairs of rollers was used in the preparation process of oriented bamboo fiber mat (OBFM). This machine removes the outside and inside layers of the bamboo culm without using chemicals [87,166]. Zhang et al. [87] got that the bonding shear strength of (OBFR) with the removal of inside and outside layers of bamboo at 20% resin content was 19.21 MPa, which is lower than the 22.5 MPa at 18% resin content of BFRC without the removal of external and internal layers of bamboo [45]. The decrease in OBFRC shear strength is attributed to the complete elimination of siliceous and waxy layers [87]. To conclude the impact of the suitable removal extent of bamboo green on BLVL bonding strength, bamboo green was removed from four bamboo bundles with different removal extents, as in Fig. 10.1

**Fig. 10:**
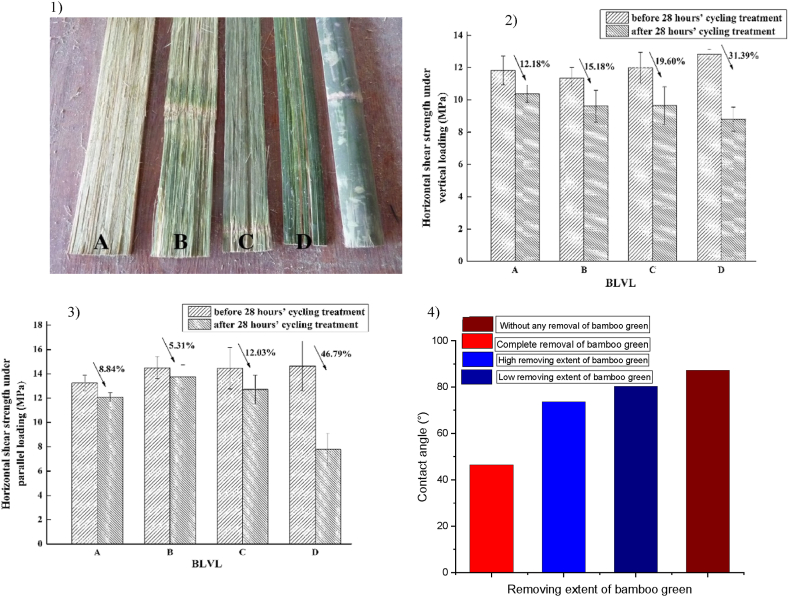
Different degree bamboo green removing extent on bamboo bundle sheets. (1) the degree of remove (a. completely removal, b. high removing, c. low removing, d. no removing). (2) Horizontal shear strength of BLVLs (2a. under vertical loading and 2b. under parallel loading). (3) The static contact angle of BLVLs [61]. (For interpretation of the references to colour in this figure legend, the reader is referred to the Web version of this article.)

**Fig. 11:**
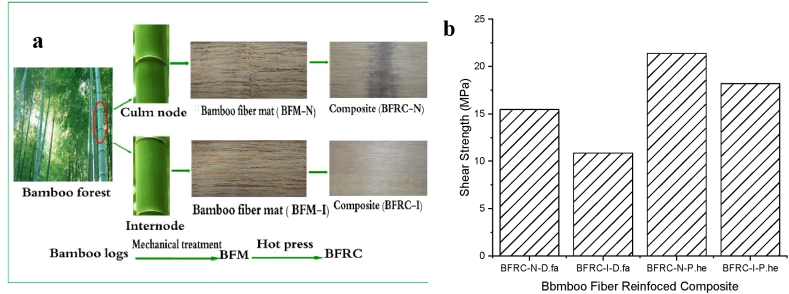
Bamboo Fiber Reinforced Composite (BFRC): (a) Production BFM and BFRC, (b) shear strength of BFRC [97].

The horizontal shear strength under vertical and parallel loading for all degrees of removal was tested before and after 28 h of heat treatment cycling, as shown in Fig. 10-2a,b. The upper the range of bamboo green removal, the greater the bonding strength, as illustrated in Fig. 10-3 [61]. A higher removal degree of bamboo green decreases the amount of SiO2 and wax; in additional, the contact angle upon the bamboo-bundle surface decreases. PF resin achieves better wettability when the contact angle is smaller. Also, a small contact angle leads to better dimensional stability of BLVLs and better bonding performance. Uniformity of the raw material is necessary when using bio-based composite in structural design as the main material. Although the engineering properties of bamboo-based compounds are satisfactory, several limitations, such as uneven stress distribution, restrict their use as a generally employed structural material [167]. Significant differences in the properties of bamboo nodes and internodes have been observed [144,168,169]. To explore the leverage of nodes and internodes on bamboo products, the mechanical properties of bamboo products were studied. Horizontal shear strength of BFRCs produced from BFMs obtained from two species (D. farinosus and P. heterocycle), with and without nodes, was investigated, as shown in Fig. 11a by Ref. [97]. The results demonstrated significant shear strength differences between BFRC-I and BFRC-N, which might be accredited toward the characteristic distinction between bamboo nodes and internodes (Fig. 11b).

**Fig. 12:**
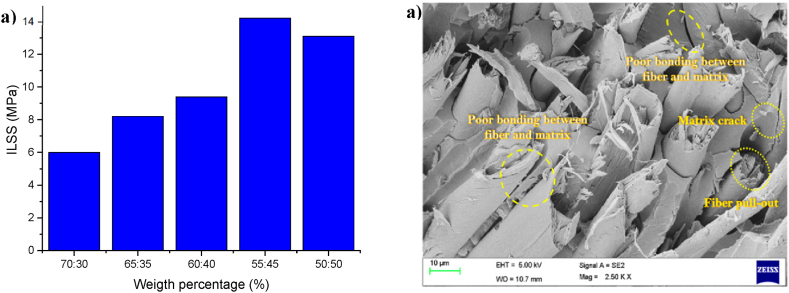
(a) Interlaminar Shear Strength of plain weave designed textile composite; (b) Surface Structure of the impact tested woven fabric bamboo composite [67].

The type of element, its size, the ratio of warp to weft, length of strand, and the part used from down to up of the culm influence the mechanical properties of bamboo products, especially bonding strength [20,67,68,71,[169], [170], [171], [172]]. Aruchamy et al. [67] examined the influence of weave arrangement and the ratio of bamboo woven fabric reinforced composite laminates on its mechanical properties. Fig. 12a demonstrates the results. The highest ILSS was recorded with 45 wt% loading at 14.2 MPa. The variation in results may be attributed to defects such as "voids, matrix fracture, pull out of fibers, rich resin, fiber delamination, and tearing," as shown in SEM Fig. 12b. Strand lengths of 70 mm, 100 mm, and 150 mm were used to investigate their impact on the engineered properties of bamboo strand boards [170]. The highest IB was recorded with a strand length of 150 mm. However, the opposite was noticed at the same length and same pressing parameters in Ref. [173]. The possible reasons may be attributed to density, amount of resin, and type of adhesive. It is concluded that there is variation in engineered properties especial mechanical properties of bamboo products with different particle sizes [68,170,171,174].

### 3.2 Age and environment outdoor condition

Variation extends not only to the 75 genera and 1250 species of bamboo, but also to engineering properties within and between bamboo species [175,176]. One of the influencing factors that impacts bonding strength is variations within and between species [62,116]. The type of bamboo species has highly effect on bonding strength, so many engineers and researchers have paid a lot of attention to this matter. Chung et al. [102] carried out a study on different OBSBs made of Chinese and Taiwanese Moso bamboo to investigate their strength properties. It was found that Taiwanese Moso bamboo OBSB had higher internal bonding properties at a density of 1.0 g/cm³, with values of 3.04 and 2.83 MPa respectively, compared to Chinese Moso bamboo. Biswas et al. [177] searched the gluability of particleboard made of B. vulgaris and B. balcooa. They concluded that the superior gluability of B. vulgaris makes it produce better particleboard than B. balcooa. Juanito et al. [8] conducted a study on arc-laminated bamboo lumber produced from two bamboo species, B. Blumeana and G. levis, to evaluate the bonding behavior of the products. The bonding performance of G. levis was found to be better than that of B. blumeana.

**Fig. 13:**
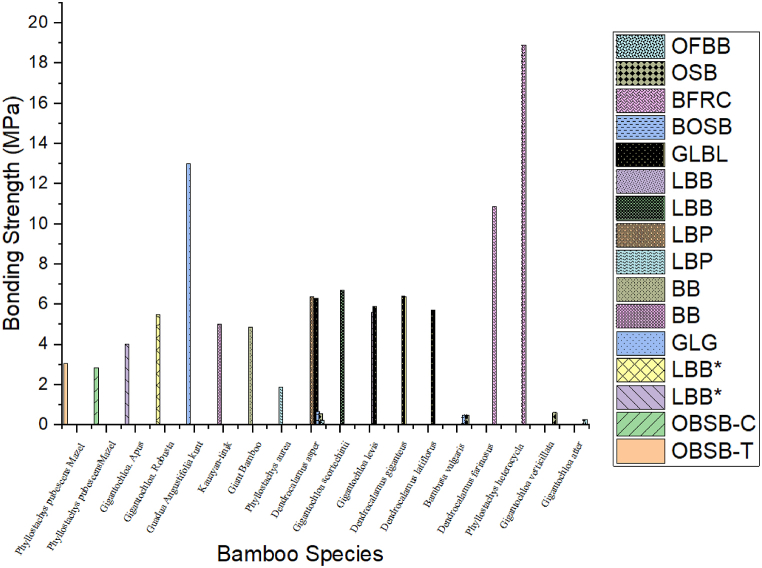
Effect of bamboo species on bonding strength. OBSB-T and OBSB-C [102]; LBB* [99]; (GLG) [118], BB [62], LBP [9]; LBB [54], GLBL; OSB [116]; BOSB [65]; BFRC [97], OFB B [180].

The bonding strength of LBB is also affected by the number of layers and bamboo species, as shown by Ref. [99]. They found that the bonding strength of G. Apus (4 MPa) was lower than that of LBB produced from G. Robusta, which was 5.47 MPa. However, the average bonding strength of LBB was lower than the samples produced from G. pseudoarundinacea, which had a bonding strength of 23.7 MPa, as tested by Ref. [178]. This difference was attributed to the use of 20 % w/w wheat flour in the adhesive. The connect shear strength of GLGB manufactured from Guadua Angustifolia Kunt with melamine-urea-formaldehyde adhesive was found to range between 12.9 and 13.1 MPa [118]. Fig. 13 shows the role of bamboo species types on the bonding strength of bamboo products. As well as, the age of bamboo species at harvest affects the gluability of bamboo products. Sun [169] studied the influence of bamboo culm age (2, 4, 6 years) on the adhering strength of bamboo-oriented strand board. The results found that the internal bond (IB) increased with increasing age, similar results were found in particleboard by Ref. [21]. Shan et al. [179] used a new artificial aging procedure to study the bonding strength of GluBam sheets. The sheets were subjected to this new aging testing method under outdoor conditions. The corresponding aging durations were 960, 480, 240, 120 days, and recently. The findings of the study revealed that aging duration is closely related to internal bonding. The longest aging duration of 960 days resulted in a residual internal bonding strength equal to only 23.4 % when compared with the specimen that was not subjected to the aging procedure. It can be clearly seen that connect strength is more sensitive to aging.

### 3.3 Density of bamboo

**Fig. 14:**
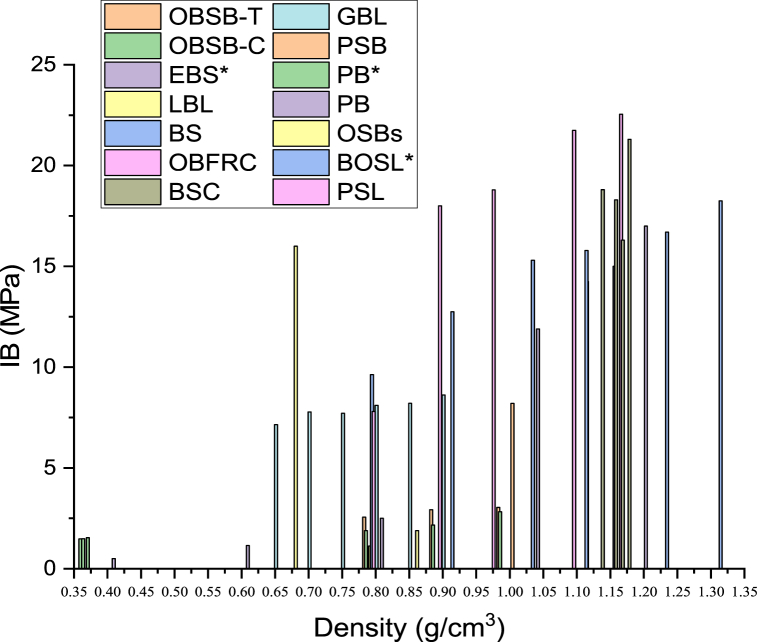
Internal bonding strength of bamboo products. OBSB-T and OBSB-C [102]; BSC [129]; BOSL* [22]; EBS [183]; LBL and BS [16]; GBL [184] PSB [185]; PB* [68]; PB [171]; OSBs [169]; OBFRC; PSL [186].

It is reported that Because of the variation in porosity from the inside to outside in the radial direction, and alongside the culm from the dowen to upper section of bamboo [181], one of the influencing factors on bonding strength is the variation in density of different bamboo strips, slivers, fiber bundles, and any segment in the culm. The resistance of pores on the bamboo culm has a negative effect on bonding strength to a certain degree. Malanit et al. [182] investigated the bonding quality of composite lumber production manufacturing with Asian bamboo (Dendrocalamus asper). They concluded that lower-density bamboo species face fewer difficulties in adhesive penetration due to thin cell walls, wide pit boles between fibers, and the size of the fiber lumen layer. Forming a better covalent bond and more compacted bamboo fibers requires enhanced density [131]. To overcome bonding strength problems and improve the appearance of bamboo scrimber, its density can be increased to 1.051.25 g/cm^3^ [130]. In another study by Hea et al. [101], wood and bamboo samples were treated using the HVEF treatment method. Fig. 14 shows the internal bonding strength of different bamboo products.

Adhesive penetration depth decreases as the density increases at the bonding interface. The hardness of bamboo scrimber layers prevents bonding from achieving as high a quality as timber layers [187]. Chung et al. [102] investigated the internal bonding strength of OBSB made from P.pubescens (Moso bamboo) with different densities (0.8, 0.9, 1.0) g/cm3. They found that the bonding strength is directly proportional to density, and all differences in IB are significant (P > 0.05). Lin and Huang [188] found that a higher IB of products is associated with higher densities. The hot pressing methods produces a higher densification of element, which in turn enhances the quality, durability and the IB of final products. The reduced internal bonding strength between the curtains of bamboo results from an increase in layer thickness, and it can cause serious damage [179]. The stability and strength of a panel's internal bond highly depend on the uniform density distribution of the panel [189]. Moreover, the shape of joints affects the bonding strength of bamboo products [190,191].

## 4 Elevated temperature

Failure models and mechanical properties of bamboo products including bonding strength are influenced by the thermal performance of adhesive, either positively or negatively, in accordance with the degree of temperature [192,193]. Frangi et al. [194] conducted a study on the bonding strength of two adhesives, namely PRF and PUR. The specimens were subjected to elevated temperatures. It was found that shear strength decreases as a result of increased temperature. Despite the temperature elevation from 20 to 70 °C, PUR adhesive retained much of its original bonding strength. PRF adhesive bonding strength decreased at 180190 °C. PUR suffered noticeably less in its bonding strength when the temperature reached more than 150 °C. In other words, PRF adhesive performed better than PUR at high temperatures. Conversely, PUR bonding was shown to withstand higher temperatures.

**Fig. 15:**
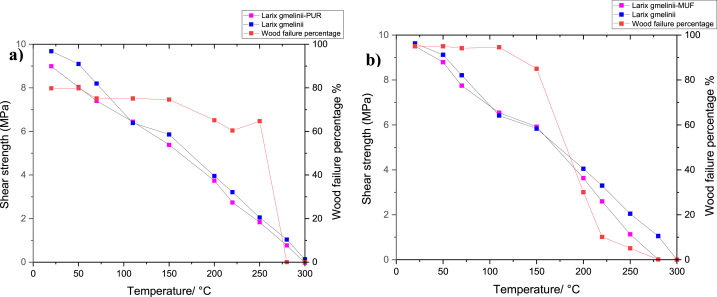
Comparison shear strength of solid wood with that of glue lines' of PRF and MUF glues under temperature [195].

Yue et al. [195] studied the elevated temperature on the bonding performance of PRF and MUF. Using either PRF or MUF adhesives, larch glulam bonding strength declined due to an increase in temperature. In the range 20 and 150 °C, shear strength of the glue-line of either adhesive was highly reliant on wood shear strength. The decrease in adhesive bonding strength between 150 and 300 °C was attributed to the fact that PRF adhesive retained its chemical structure undamaged at 220 °C, while MUF chemical components suffered significant damage. Linear deterioration of PRF and MUF bonding performance is attributed to increased temperature, as shown in Fig. 15a and b. This figure also shows the WFP at raised temperatures. At room temperature, PRFs' shear strength was less than the shear strength of solid wood. However, in same environment, MUFs’ shear strength exhibited excellent bonding performance that was similar to the shear strength of solid wood.

Clau et al. [196] conducted a study on beech glue line and shear strength of MUF beech and beech PRF, finding that beech has better bonding strength than larch. Both test samples of beech demonstrated superior bonding strength compared to the larch experimental fitting model in Ref. [195]. George et al. [197] investigated the comparative creep of glulam wood adhesive. The results showed that MOR of RPF was 2250 MPa at 25 °C, but then decreased after 175 °C. Prolonged burning time of resorcinol resin adhesive RF led to a decrease in MOR and MOE, with the decrease in MOE and MOR connected to the residual area [198]. Without regard to temperature, the stiffness of prepolymer films, in additional the shear strength property of bonded wood joints, meaningfully improved with a greater content of urea stiff parts [199]. Silva et al. [200] studied the mechanical characteristics of wood composites produced using different glues (Redux 326 film, Redux 326 paste, Supreme 10HT, and Hysol EA 9359.3) at elevated temperatures ranging from 55 to 200 °C. Mechanical tests of shear and tensile strength demonstrated that the strength and stiffness displayed a linear relationship as temperature increased. According to all the studies mentioned above, the shear strength of different adhesives in different products demonstrated a linear relationship with increasing temperature.

## 5 Effect of manufacturing procedures on bonding strength of bamboo products

Since the 1980s, freshly engineered materials and manufacturing techniques have allowed for the creation of prefab bamboo dwellings made from laminated bamboo boards, veneers, panels, bamboo scrimbers, and so on [17]. These products are referred to as engineered bamboo and have been processed in various ways to create regular, construction materials with straight edges from irregular, circular canes. During manufacturing, various parameters can influence mechanical properties, including bonding strength, such as layers’ arrangement, type of treatment, clamping pressure, time under pressure, type of pressing, temperatures, surface characteristics, loading direction, etc.

### 5.1 Layered structure

**Fig. 16:**
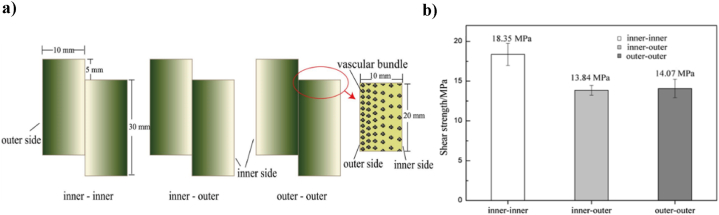
Schematic diagram of glued bamboo strip samples [201]: a) A configurations on adhering strength of gluing strips and b) connect strength of final products concentrated on the inside face of bamboo.

A necessary requirement for the quality of engineered bamboo (EB) renewable products is the bonding strength properties, which are influenced by many parameters including the structure of the layer and the characteristics of the layers' surface, such as roughness and softness, which can either positively or negatively affect bonding properties. For better mechanical behavior of engineered bamboo, bamboo hybrid bonding strength must be improved and upgraded [187]. Many researchers have studied the mechanical bonding properties. In a research by X. Lu et al. [101], the samples were arranged into three model: bamboo outer-bamboo outer, bamboo outer-bamboo inner, and bamboo inner-bamboo inner. The arrangement II < OI < OO achieved a great improvement in the bonding strength rate. The highest bonding performance was achieved by the type OO, which increased the bonding strength by 37 % and had a positive influence on mechanical properties, in same time the lowest wood failure ratio. That is attributed to densification and decreased penetration depth in OO type. Yu [201] studied the effect of assemble configurations on adhering strength of gluing strips (Fig. 16a). The results demonstrated that the parenchyma cells have a significant contribution to the connect strength of final products concentrated on the inside face of bamboo (Fig. 16b). Chaowana et al. [23] used three combinations of laminated bamboo lumber (LBL) layered structures to evaluate the bonding strength and gluability of LBL. The layered structure was arranged OO, OI, and II. The glue of the layered structure was found to be an important factor for LBL bonding strength. It was also found that bonding strength is stronger in the I-layer than in the O-layer. The results of the above study were confirmed by Ref. [93] who tested bamboo zephyr mats, and by Ref. [39], who tested plybamboo. Moreover [202], tested flattened bamboo-based glulam. A weak bonding strength and surface wettability of laminated bamboo lumber with a higher contact angle, when using outer layer of bamboo culm [23]. All the above researches prove that the location of bamboo species within the culm influences the bonding strength of final products.

**Fig. 17:**
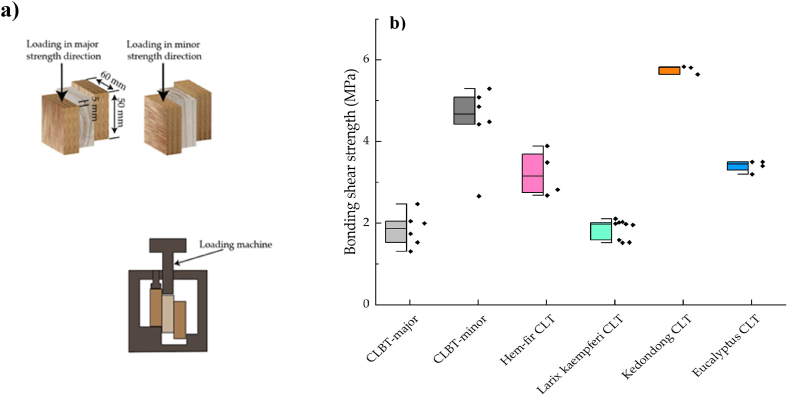
(a) Schematic, sampling and set-up test of shear bonding and CLBC [205]. (b) Bonding shear strength of CLBT compared with CLT [205], Larix kaempferi CLT [211]; Hem-fir CLT [124]; Eucalyptus CLT [212]; Kendondong CLT [204].

**Fig. 18:**
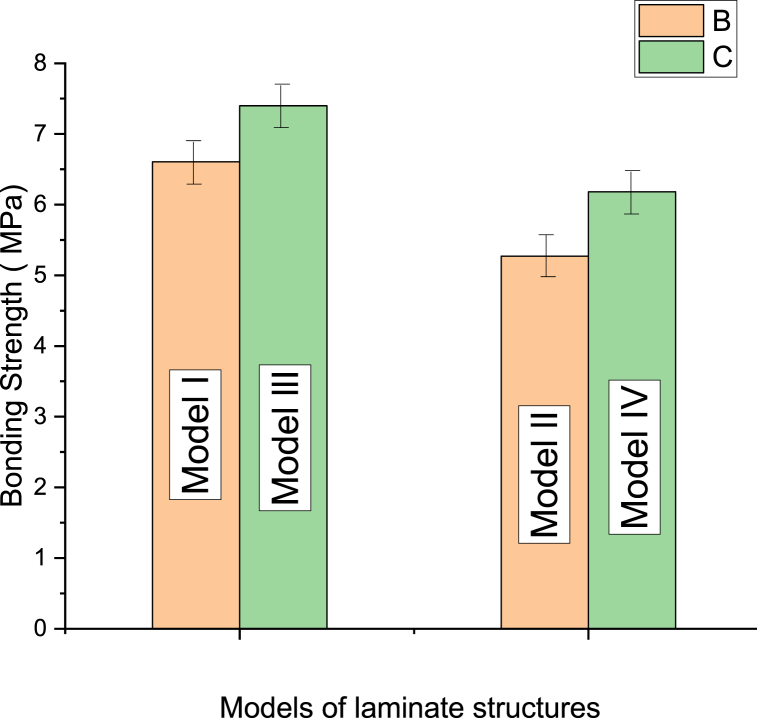
Bonding strength of four models of BWHC [105].

Bonding strength of composite structural is influenced by the direction and type of layer configuration [203]. Many academic and business ventures pay a lot of attention to developing and improving bamboo renewable products. One of the pioneers is an institute in China that seeks to develop and produce bamboo-wood composite products [33,[204], [205], [206], [207], [208]]. The bonding durability of these products has a optimistic effect on engineering properties, and it has been tested in many studies. To assess and evaluate the durability and bonding quality of bamboo-wood CLT, adhering tests were carried out by Ref. [35]. 2.38 MPa of CLT adhering laminar strength was found, which is the same strength as hem-fir CLT that was tested by Ref. [209]. When bamboo-wood composite was subjected to dry conditions, the wood and block shear strength failure rates were 70.6 % and 2.07 MPa, respectively, which were less than WFP of hem-fir CLT. The failure percentage of wood and block shear tested under vacuum pressure soaking then drying conditions was found to be lower than hem-fir CLT, as in the previous study. 6.8 % average delamination CLT composite with PUR adhesive. It demonstrates that the bonding durability of bamboo-wood composite CLT is better than hem-fir CLT. Yang [205] investigated the durability and bonding quality of CLBT. In parallel and perpendicular were loaded (Fig. 17a). Two types of adhesive and three bonding pressures were applied to the samples. A significant impact was noticed with types adhesives and loading direction, however, the pressure had no influence on bonding quality and WFP. The bonding strength was compared with different CLT products (Fig. 17b). The shear strength of amabilis fir and western hemlock was higher than bamboo in parallel direction [210], however, opposite found by Ref. [209]. The BSS of both bamboo-wood CCLT and hem-fir CLT was less than the wood itself in parallel direction. Chen et al. [105] used four models of laminate structures to evaluate their leverage on the bonding strength of BWHC. A higher BWHC bonding strength in parallel than perpendicular was obtained, as shown in Fig. 18. The veneer horizontal layer located between the layers of bamboo chopstick greatly enhanced the BWHC bonding strength, as supported by SEM observation. The BWHC bonding strength value was also tested three times and found its higher than the bonding strength of chopstick plywood [105].

**Fig. 19:**
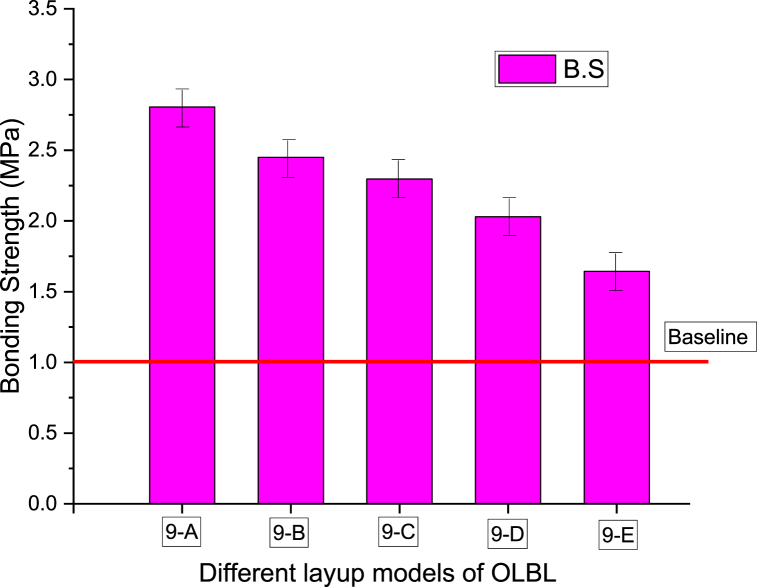
OLBL with different layup models. The required value of bonding strength that agreed in standard ‘‘Plybamboo form” [213] represents by the baseline [106].

Bonding capacity of OLBL with different layup models has investigated by Ref. [106]. The samples were fabricated in nine structural layers using radial and tangential bamboo curtains glued with phenolic impregnatable adhesive paper. The five groups' bonding strength is shown in Fig. 19. Groups 9-B, 9-C, 9-D, and 9-E have the highest COV. This can be attributed to the gaps in the radial bamboo curtain that led to variations during hot pressing. The random sampling of the tested specimens caused small variations in porosity, causing in high distinctness in bonding strength in the same group. All five groups have bonding strengths that conform to "plybamboo form" standard requirements [213]. The results above demonstrate that the layup and direction of bamboo laminate or bundle fiber significantly affect the bonding strength of bamboo products.

### 5.2 Moisture content

Mechanical properties of bamboo are dependent on moisture content, which is a very significant physical property. For a material to have high bond quality, moisture content must be closely controlled. Different countries have their standard requirements for average moisture content. For example, the average moisture content for wood interior applications ranges between 6 and 11 % [214]. The hydrophilic nature of bamboo fiber complicates the process of maintaining bamboo laminates at a lower moisture content; as a result, the recommended method before lamination of a specimen is oven drying. Different studies have recorded varying moisture content in the initial and test stages, from 8 to 16 %. Lee et al. [153] investigated the influence of initial moisture content on bamboo strength. They found that the mechanical properties of bamboo were not affected by moisture content. Nonetheless, dimensional stability deteriorated due to moisture content. Lim [215] reported that for light flooring and average traffic conditions, the dry shear of the glue line should be more than 1.42.0 MPa. Because bamboo flooring materials are still in their infancy, no load or state standard or regulation has been set. As a result, wood species standards are borrowed to study and regulate the properties of bamboo floorboards [62]. To achieve good bonding for bamboo and bamboo products, the moisture content should be 8%12 % [216].

### 5.3 Processing methods and procedures

For durable products, manufacturers subject bamboo products to preservation treatment procedures during the manufacturing process. Chemical bleaching and hydrothermal caramelization are two of the most common processing methods in the industry of bamboo composites. The effects of these two processing methods have been the focus of many studies [39,42,217]. It is found that compared to treated laminates, untreated laminates have higher shear strength. The bond quality of Gigantochloa scortechinii laminates is higher than Dendrocalamus asper, independent of bleaching systems [218]. The shear strength of the unbleached G. scortechinii was 4.60 MPa in dry condition and 3.47 MPa in CBR, compared to 4.40 MPa in dry and 2.11 MPa respectively for D. asper. However, the failure percentage of the dry and CBR wood was 100 %. Different test results on laminated bamboo demonstrated that the bleaching processing method is better than the caramelizing method because it improves surface properties for bonding [85,218]. This evident improvement is attributed to the modification of the chemical lignin content, which produced higher surface energy in comparison to caramelizing [85,219]. Many studies have evaluated the effect of different common preservation treatments such as dry heat treatment [107,109,220] and chemical treatment [40,42,217] on the bonding and adhesion properties of bamboo laminates as happened with treatment in other structural composite materials [221]. All studies concluded that treatment has a negative effect on glue line bonding strength and wettability, with a reduction ranging from 15 % to 70 % [85]. PF bonded laminated bamboo boards suffered a decrease in bonding strength when glued skin to skin after oil heat treatment, whereas it showed a significant improvement when glued pith to pith before and after being heat-treated [220].

**Fig. 20:**
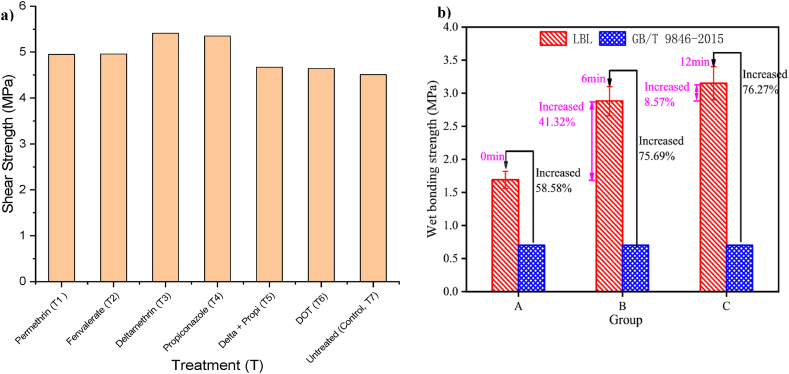
(a) Effect of preservative on shear strength of engineered bamboo boards [62], (b) the influence of O_2_ Plasma modification with different time on bonding strength of LBL, the code group indicate to: group A: untreated, group B: 6 min treated, group C: 12 min treated [78].

HVEF is a promising technology treatment that has significant effects on the performance of the surface and interface of different materials [222]. Qian et al. [223] studied the effect of HVEF on the mechanical properties of LVL. The mechanical performance of LVL was highly improved because the HVEF method increased the shear strength limiting value. The study concluded that the HVEF processing method significantly improved the different bonding types of bamboo. UV irradiation is another processing method that has been shown to improve wettability and surface bonding of bamboo [224,225]. The influence of using preservative treatment and variant glue types on engineered bamboo board bonding strength was conducted by Ref. [62]. For housing high-end products such as windows and doors, the best-recommended preservative combination is PVAc deltamethrin. Fig. 20 shows the variation in bonding strength of some bamboo products using different preservative treatments (Fig. 20a) [62]. Wu et al. [78] studied the development of bonding strength of LBL using O2 Plasma treatment at different times and compared it with untreated samples as a control. The results showed enhancement in wetting surface and penetration of resin on the bamboo surface with O2 plasma, but its effectiveness weakens over time. The LBLs' bonding strength increased by 58.58 % when compared with ordinary plywood, as seen in Fig. 20b.

**Fig. 21:**
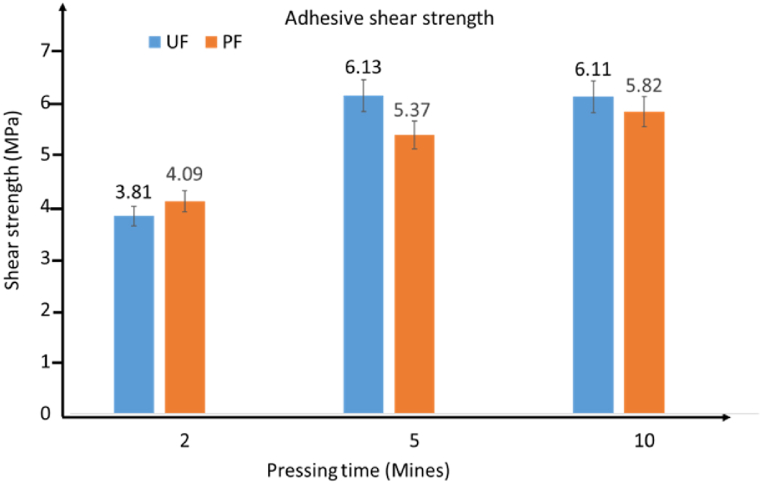
Phenol formaldehyde (PF) and Urea formaldehyde (UF) adhesives shear strength at different times (25 min) [60].

The unbalanced surface of bamboo zephyr mat, especially thin boards, is attributed to hot processing. This creates many close spaces among the elements which can decrease the bonding strength between these elements. To overcome these limitations, the hot-press pre-treatment method was used on bamboo zephyr mats [91]. Wood or bamboo fibers treated in boiling water decreases interfacial bonding when the fiber surface is not provided with glue [226]. Water penetration is also another problem caused by the lack of glue in the repeated treatment process [131]. Wang et al. [227] investigated the bonding strength of chopstick plywood that was processed using hot treatment. The gaps between chopsticks played an important role in mechanical properties. Ahmed et al. [60] assessed the effect of different pressing durations [[2], [3], [4], [5], [6], [7], [8], [9], [10]] minutes on bamboo slabs that were glued using two different types of adhesives, as shown in Figure 21. Five minutes was the best pressing duration for the Urea formaldehyde bonded sample, while 10 minutes was chosen because they presented the highest shear strength among all samples.

The another study by Mingjie et al. [228] was conducted to investigate the effect of IFVs drying conditions on the bonding strength of bamboo scrimber. A perpendicular method was used to evaluate the effect of many factors on bamboo scrimber bonding strength. The relationship between precuring rate and moisture content of PF resins on IFVs applied on different drying conditions and bamboo scrimber bonding strength. The effect of each factor on bonding strength was from the highest to the lowest: drying temperature > solid content of PF resin > drying time. The study results showed a close relationship between bamboo scrimbers’ bonding capacity and precuring time. The best bonding capacity has been achieved when the solid content was 60 °C and 1.5 h drying conditions, and 20 PF resin. The bonding capacity of bamboo scrimber reached 2.95 MPa, and 12.30 % of the PF resin as precuring rate on the IFVs surface. Impregnated fluffed veneers result from an improper drying process, decreasing both the properties of bamboo scrimber [[229], [230], [231]] and its bonding strength. Microwave-assisted curing is another common method. Zheng et al. [110] investigated PSB glue strength cured with a microwave-assisted method. The study concluded that the faster the microwave-assisted curing, the rougher the surface and the higher the bonding strength of PSB.

Interface adhesion can suffer weakness or debonding due to many complex factors such as the hydrothermal aging process because LBC hydrothermal aging is one of the material hydrolysis components and it can weaken the interface [232]. Due to high pressure and fluffing, the crack of OBFM, Lumina, vessel, and parenchyma intercellular space was filled with resin. The resin and the bamboo ingredients formed a bonding interface enhancing weak intercellular layers [87]. Glue bonding of bamboo quality can also be influenced by the uniformity of the material and processing methods applied during manufacturing. These two important factors attract the attention of many researchers and specialists. The better bonding performance of bamboo fiber is also the result of better uniform material [233]. A statistical analysis method can be used to monitor the influence of assembling series on the bonding behavior of bamboo bundle wood veneer laminated lumber.

**Fig. 22:**
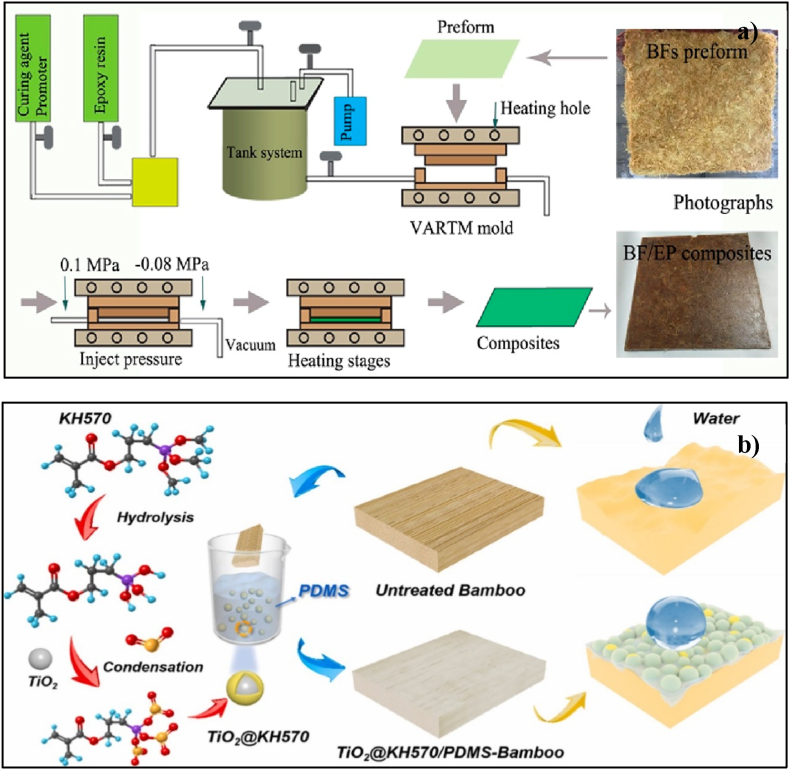
(a) Schematic fabrication of BFs and BFs/EP by resin transfer molding (RTM) [243]; (b) schematic preparation of TiO2@KH570-bamboo [244].

Recently, many physicochemical techniques have been employed to enhance the qualities of bamboo products and broaden their application domains, as discussed above. Given their antibacterial capabilities and the hydrophobic microstructure caused by surface self-assembly, nano-sized inorganic material modification is considered more beneficial than the previously described approaches for improving the qualities of biomass materials [234,235]. However, when combined with biomass, inorganic elements typically endow decorative composites with additional, unique properties, such as optics [236,237], electricity [238,239], and magnetism [240,241], thereby extending their range of applications and increasing their added value. The characteristics and performance of the resulting composite materials are largely determined by the bonding mechanisms within the polymeric matrix, the bamboo fiber reinforcement, and their interface. It is demonstrated the surface modifications made to enhance interface bonding in bamboo fiber-based composites Fig. 22a and b. Moisture content and lignin are two factors that tend to weaken the interfacial adhesion between the matrix and reinforcement phases, resulting in the production of defects and a loss of strength that degrades the quality of the composite materials. Various chemical treatments have been applied to achieve optimal characteristics of bamboo reinforced composite materials by enhancing interfacial bonding/adhesion [242].

The higher the defibering times, the higher the bamboo bundles specific surface area, which enables the adhesive to go deeper and improve products bonding strength. Bonding performance of bamboo scrimber is also determined by another vital manufacturing process that is dipping [156]. The direct improvement of PF resin penetration and bamboo scrimber bonding strength was the result of fluffed veneer [219,220]. A possible reason for that is the sufficient distance between bamboo fiber after their brooming facilitating the infiltration and parceling of PF resin well. Consequently, obtaining a perfect bonding interline of BLVL and enhancing the dimensional steadiness of the board to a large size [245].

### 5.4 Surface characterizes

**Fig. 23:**
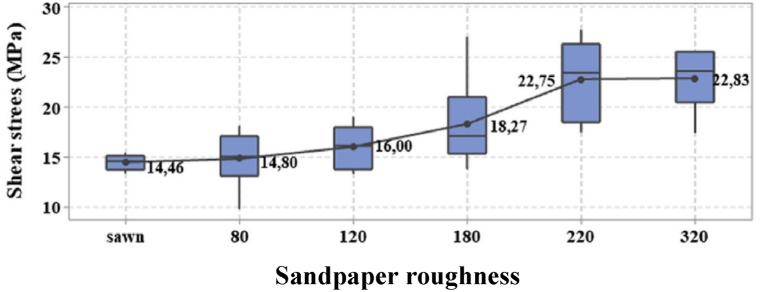
Shear stress via sandpaper roughness [64].

Different processing methods of bamboo cause many desired and sometimes undesired modifications on surface characteristics, strength, and bonding. The impact of outward appearance coarseness on the bonding capacity of bamboo and wooden material was the focus of many researchers and studies [82,216,[246], [247], [248]] with a similar impact detected in identical structural materials [249]. Carrasce et al. [64] tested the leverage of the coarseness of Glubam on the bonding capacity. Sandpaper was used to conclude the degree of roughness. The influence of roughness on bonding capacity is presented in Fig. 23 [64]. The results demonstrated that there was no change between shear stress at the surface without sandpaper and with sandpaper 80°. In addition, the roughness degree between 220 and 320 showed a small increase in shear stress. It concluded that the degree of roughness between 80 and 220 causes a considerable increase in bonding strength that can reach a 50 % increment, which is significantly beneficial to bamboo elements and produces Glubam. However, the maximum shear strength of bamboo scrimber was reached at 120 mesh sanding [250]. Sogutlu [251] investigated the leverage of wooden materials outward appearance roughness found on bonding capacity. Jimenez et al. [8] used two kinds of bamboo to find out the correlation between surface coarseness and bonding capacity. Laminate bonding performance was found to be influenced by surface roughness. Mechanical and physical properties of particleboard prepared from soft and rough particles were reconnoitered by Karlinasari et al. [20]. The results presented that internal bonding increased with the decreasing of particle size; however, the opposite was noticed with other mechanical properties, similar results found in Refs. [174,252].

A noticeable variation in wettability between both the inner and outer surface of the culm wall has been reported in many studies, which clearly affects the material's bond strength [23,109,112,253,254]. The valuable contributions of researchers on surface wettability of varied supplies using CA examine have improved BFRc permeability and aided in the study of interfacial bonding behavior. A comparison was made between the contact angle of a single bamboo viscose fiber and that of terylene fiber. Measurements were taken at varying environment. Humidity and temperature have an important leverage on the contact angle of natural fibers, for example a bamboo, due to their distinct structures and chemical compositions. In contrast, as a synthetic chemical fiber, ethylene's contact angle exhibited little variation due to temperature changes, but no change was observed in response to humidity changes [255].

The spread rate of liquid and how fast it can spread and wet a bamboo product is determined by the degree of wettability of bamboo. Wettability of bamboo and adhesive influences bonding characteristics; wettability is measured by contact angle. An increase in wetting narrows the contact angle, consequently resulting in poor bonding products [217,256]. Bamboo strips processed using LCSP chemical material treatment had the highest contact angle among all other chemically treated materials used in the test [217]. The bonding process and the joint's glue bond quality are both considerably influenced by the surface [257]. The rougher the surface, the better the bonding strength, while the smoother the surface, the poorer the bonding strength [258]. In addition, treating the surface before adhesive application is recommended to achieve better bonding strength [259]. The following pretreatment methods can also achieve good bonding strength: resin pre-coating (RPC) treatment [258], physical methods such as atmospheric pressure [260], laser ablation [261], sandblasted surface [262], and grit-blasted surface treatments [263].

## 6 Challenges and future prospects

The current limitations in the bonding quality of bamboo composites necessitate rigorous and expansive research. Priorities for future studies should include.

- There's a critical need to maximize the recovery of bamboo resources while concurrently improving the bonding quality of bamboo-based materials. This encompasses a holistic approach, integrating sustainable harvesting, processing, and utilization techniques.
- The development and establishment of comprehensive standards for bond qualification testing of bamboo products is imperative. These standards should align with existing protocols for wood products, thereby ensuring consistency and reliability in quality assessment.
- There is a need for a focus on expanding and refining surface treatment methods is crucial. This would enhance interpenetration, reduce decay, and improve overall bonding quality, thereby extending the longevity and durability of bamboo-based composites.
- The innovation in resin technologies, aiming to achieve a balance between high bond strength and cost-effectiveness, is essential. Tailoring resin formulations to meet specific requirements of bamboo composites can revolutionize their application in construction.
- To focus on enhancing the long-term durability of bamboo composites, particularly in resisting environmental factors like moisture and temperature fluctuations.
- To develop methods to scale up the production of bamboo-based composites while ensuring quality and performance for construction needs.
- To conduct research to identify and optimize environmentally sustainable adhesives that improve the bonding strength of bamboo composites.
- To investigate the global market potential for bamboo-based composite materials in the context of the growing sustainable construction industry.

Despite its potential, several challenges hinder the widespread adoption of bamboo composites in construction.

- It is recommended to achieve high bonding strength at a low cost remains a significant challenge. This requires innovative approaches in material science to develop cost-effective bonding agents without compromising strength.
- The absence of universally accepted standards and codes for bamboo products is a major impediment. This gap hinders the assessment, comparison, and certification of bamboo-based materials.
- To develop standardized methods to minimize variability in bamboo quality, ensuring consistent performance in composite materials.
- To Focus on developing comprehensive testing protocols to better assess and enhance the long-term durability of bamboo composites in various environmental conditions.
- To conduct in-depth research to find and optimize eco-friendly adhesives that enhance the bonding strength of bamboo composites without compromising sustainability.
- To investigate and implement innovative production techniques that can scale bamboo composite manufacturing efficiently, maintaining quality at higher volumes.
- To work towards creating and refining building codes and standards specifically for bamboo-based materials, facilitating their broader acceptance in construction applications.
- To analyze the economic challenges in producing bamboo composites, aiming to make them competitive with traditional construction materials.
- The varied characteristics of bamboo species across different geographical regions add complexity to standardization and application. Understanding and categorizing these variations are crucial for effective utilization.
- There are several environmental and biological factors such as corrosion, insect infestation, and other environmental influences can adversely affect engineered bamboo construction. These issues can lead to cracks and deterioration of the mechanical properties of bamboo products. Traditional reinforcement methods, like increasing the cross-sectional area of components, often negatively impact the resin's interpenetration and bonding effectiveness.

In brief, bamboo-based materials have already shown immense potential across various domains. With focused research and innovation, particularly in the areas of bonding strength and material standardization, the future prospects for bamboo in sustainable construction, including applications in building construction, bridge building, concrete composite construction, and high-strength materials, are exceptionally promising. This underscores the critical need for continued research and development in this field to harness the full potential of bamboo as a sustainable construction material.

## 7 Conclusions

In conclusion, this comprehensive review has systematically examined the multifaceted factors that significantly influence bonding strength performance in bamboo-based construction applications. Drawing upon a wealth of knowledge from the extensive body of literature available globally, we have synthesized key insights into the critical determinants of mechanical resilience and long-term serviceability in structural applications involving engineered bamboo materials. This critical analysis has underscored the paramount importance of adhesive selection in achieving optimal bonding performance. Among the myriad options available, phenol-resorcinol-formaldehyde (PRF) adhesives have emerged as the quintessential choice for constructional bamboo applications, particularly when resin content falls within the range of 10 %18 %. This optimal adhesive selection fosters the development of the most formidable interfacial bonds, ensuring the structural integrity of bamboo-centric products.

Furthermore, this review has illuminated the profound impact of chemical constituents within bamboo culms on interfacial bonding. The escalated removal of these constituents was found to significantly enhance bonding properties, emphasizing the need for meticulous material quality control and processing techniques to harness bamboo's full potential. Among the diverse bamboo species scrutinized, P. heterocycla and G. angustifolia kunt have stood out as exemplifying superior interfacial bonding capabilities. However, it is important to acknowledge that the integrity of these bonds may diminish over extended durations under sustained load conditions, necessitating further research into enhancing long-term performance.

Temperature resistance has been identified as a crucial consideration in bamboo-based construction, with melamine-urea-formaldehyde (MUF) adhesives displaying commendable bond strength but exhibiting structural degradation when exposed to temperatures exceeding 150 °C. In contrast, PRF adhesives have proven their mettle by retaining robust interfacial bonding even under elevated thermal conditions, making them a reliable choice for applications subject to heat stress. From a processing perspective, our findings have demonstrated that the bleaching method surpasses the caramelizing technique in augmenting bamboo's surface amenability to bonding. This outcome is primarily attributed to the modulation of bamboo's inherent lignin content, resulting in enhanced surface energy dynamics. In addition, the roughness of surfaces has been shown to correlate positively with bonding capabilities, emphasizing the significance of surface preparation in achieving optimal performance.

In conclusion, this review not only consolidates the existing wealth of knowledge on bonding strength performance in bamboo-based construction but also smooths the way for upcoming research and innovation in this domain. As the global construction industry increasingly embraces sustainable and resilient materials, the insights presented here will serve as a valuable resource for engineers, researchers, and practitioners striving to unlock the full potential of bamboo in structural applications. The synthesis of this extensive body of knowledge will undoubtedly contribute to the advancement of sustainable construction practices worldwide, ultimately shaping a more resilient and environmentally responsible built environment. Furthermore, several detailed conclusion points were highlighted below.

− The studies have proved that MUF and PRF adhesives have optimum a chance for bamboo products to use in an exterior and interior structural applications.
− The best optimal resin content for production of bamboo is between 10 and 18 %.
− The chemical content showed negatively on interfacial bond. That caused a weak in properties of products and their potential on structural applications.
− The type of bamboo species, area and age under outdoor conditions significantly demonstrated on bonding strength.
− MUF adhesive showed better connection strength that was alike to bonding strength of solid wood than PRF at low temperature but at high temperature oppositely noticed. The decrease at high temperature attributed to adhesive attained its chemical structure damaged.
− A lot of studies concluded that treatment have a negatively affected on glue line bonding strength. As well as the studies demonstrated that bleaching processing method is better than caramelizing method on bamboo products because it improves surface properties for bonding. This is credited to the amendment of the chemical lignin contented, that produced higher surfaces energy.
− The rough surface has bonding strength better than smoother. In addition, treatment of the surface before adhesive application is recommend.

In light of the principal findings discerned within this investigation, several promising areas of inquiry are proffered as salient research subjects for subsequent investigation. These prospective lines of study are presented herein with the aim of fostering an expanded and nuanced comprehension of the multifaceted domain of bamboo and its engineering properties, particularly with respect to the intrinsic interplay with its adhesive properties, such as the interlaminar shear strength (IB). The ensuing recommendations are couched in the rubric of scientific academic prose.

− The current study emphasizes the substantive impact of lay-up mode and bamboo species on the failure behavior and IB of bamboo products. It is incumbent upon the scientific community to embark on more extensive research endeavors aimed at elucidating the intricate interplay of these variables and their subsequent influence on the overarching mechanical properties of bamboo-based products.
− The investigation brings to the fore the underdeveloped understanding regarding the effects of environmental factors, particularly weather conditions, on the durability of bamboo products. To glean comprehensive insights into the matter, it is imperative that further studies be conducted to meticulously evaluate the influence of various environmental phenomena on the mechanical attributes of bamboo products, including IB.
− Given the notable variability in bamboo species, it is crucial to embark on focused investigations into the mechanical properties and adhesive characteristics of different bamboo species. Comparative studies encompassing a diverse array of species could offer valuable insights into the potential for species-specific utilization in various engineering applications.
− The study demonstrates a discernible augmentation in bamboo density with progressive aging. This outcome engenders a pressing exigency for more exhaustive investigations into the ramifications of bamboo age on an array of mechanical properties, notably encompassing the pivotal dimension of Interlaminar Shear Strength.
− The examination elucidates that the mechanical attributes of bamboo products are intrinsically tied to the dimensions of constituent elements. To attain bamboo products with optimally configured element sizes, it is imperative to engage in further systematic inquiries that encompass diverse treatment temperatures and composite element size distributions.
− While this study primarily focused on mechanical properties in relation to age and element size, there exists a paucity of research into the longitudinal versus radial mechanical attributes of bamboo. Further studies should be conducted to ascertain the variations in properties along these distinct axes, elucidating their implications for engineering applications.
− The growth conditions of bamboo, including factors such as soil type, climate, and altitude, can significantly impact its mechanical properties. Future research endeavors should seek to comprehensively explore the influence of diverse growth conditions on bamboo's mechanical characteristics and adhesive properties.
− In the context of bamboo-based composites, it is imperative to delve deeper into the incorporation of other reinforcing materials, such as natural fibers or synthetic additives, to enhance mechanical properties. Investigating the synergy between bamboo and these materials offers potential for tailored composite solutions with superior performance.
− Bamboo is often promoted for its sustainability and eco-friendliness. However, empirical assessments of its ecological impact, from bamboo cultivation to product fabrication, are limited. Future research should encompass life cycle assessments to quantify and validate the environmental benefits of bamboo-based materials.
− Building upon the age-related findings, longitudinal studies tracking bamboo's mechanical properties as it ages and weathers in real-world conditions are necessary. Such investigations can provide insights into the long-standing resistance and performance of bamboo products.
− The application of advanced testing techniques, such as non-destructive testing and microstructural analysis, can offer a deeper understanding of bamboo's mechanical behavior. Integrating these techniques into future research can yield more comprehensive and nuanced insights.
− Establishing standardized testing protocols and codifying design guidelines for bamboo-based products can facilitate their widespread adoption in engineering and construction. Collaborative efforts between researchers, industry stakeholders, and regulatory bodies are pivotal in this regard.
− Encouraging interdisciplinary collaboration between material scientists, engineers, ecologists, and architects can foster a holistic approach to bamboo research. Such collaborations can lead to innovative and sustainable design solutions that leverage bamboo's unique properties.

In summary, the outcomes of this study not only furnish valuable insights into the mechanical and adhesive attributes of bamboo but also pave the way for an enriched landscape of scientific inquiry. These recommendations delineate prospective avenues for further exploration and discovery, thereby contributing to the broader corpus of knowledge surrounding bamboo and its manifold applications in engineering and construction disciplines.

## CRediT authorship contribution statement

**Yousef Sewar:** Writing – original draft, Software, Resources, Methodology, Formal analysis, Data curation, Conceptualization. **Mugahed Amran:** Writing – review & editing, Validation, Supervision, Methodology, Funding acquisition, Formal analysis, Data curation, Conceptualization. **Siva Avudaiappan:** Writing – review & editing, Validation, Resources, Formal analysis, Conceptualization. **Yaser Gamil:** Writing – review & editing, Validation, Resources, Formal analysis, Data curation. **Raizal S.M. Rashid:** Writing – review & editing, Visualization, Validation, Resources, Data curation.

## Declaration of competing interest

The authors declare that they have no known competing financial interests or personal relationships that could have appeared to influence the work reported in this paper.
